# Bis[μ-*N*′-(adamantan-1-ylcarbon­yl)-2-oxidobenzohydrazidato(3−)]tetra­pyridine­trinickel(II) dimethyl­formamide monosolvate monohydrate

**DOI:** 10.1107/S1600536812013396

**Published:** 2012-04-18

**Authors:** Han-Chang Wei, Wan-Yun Huang, Xiang Zhou, Meng Shi, Fu-Pei Liang

**Affiliations:** a Nanning Prefecture Education College, Nanning, Guangxi 530001, People’s Republic of China; b Key Laboratory for the Chemistry and Molecular Engineering of Medicinal Resources (Ministry of Education of China), School of Chemistry and Chemical Engineering, Guangxi Normal University, Guilin 541004, People’s Republic of China

## Abstract

In the title trinuclear Ni^II^ compound, [Ni_3_(C_18_H_19_N_2_O_3_)_2_(C_5_H_5_N)_4_]·C_3_H_7_NO·H_2_O, three Ni^II^ cations are bridged by two *N*′-(adamantan-1-ylcarbon­yl)-2-oxidobenzohydrazidate trianions. The central Ni^II^ cation has a distorted octa­hedral N_4_O_2_ coordination environment where a reverse torsion occurs between the two bridging ligands, whereas the two Ni^II^ cations on the sides each adopt an N_2_O_2_ square-planar coordination. Weak intra­molecular C—H⋯O and C—H⋯N inter­actions help to stabilize the mol­ecular structure. In the crystal, the lattice water mol­ecule links with the Ni^II^ complex and dimethyl­formamide solvent mol­ecule *via* O—H⋯O hydrogen bonding.

## Related literature

For the use of *N*-acyl­salicylhydrazide in the construction of polynuclear complexes and metallacrown structures, see: Liu *et al.* (2008 ▶); Moon *et al.* (2006 ▶); Qin *et al.* (2011 ▶); Wang *et al.* (2005 ▶). For applications of complexes with *N*-acyl­salicyl­hydrazide ligands, see: Alexiou *et al.* (2003 ▶); Li *et al.* (1996 ▶); Zeng *et al.* (2007 ▶); Zhou *et al.* (2010 ▶). For related structures, see: Lin *et al.* (2007 ▶); Meng *et al.* (2007 ▶); Xiao & Jin (2008 ▶); Yang & Lin (2005 ▶). 
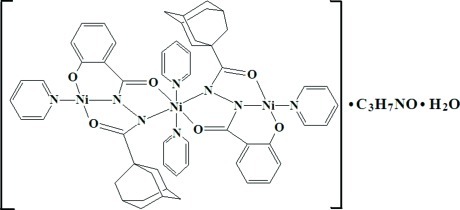


## Experimental

### 

#### Crystal data

[Ni_3_(C_18_H_19_N_2_O_3_)_2_(C_5_H_5_N)_4_]·C_3_H_7_NO·H_2_O
*M* *_r_* = 1206.35
Triclinic, 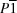

*a* = 14.3496 (8) Å
*b* = 14.8499 (9) Å
*c* = 15.2256 (9) Å
α = 62.061 (1)°
β = 72.261 (1)°
γ = 85.202 (1)°
*V* = 2723.3 (3) Å^3^
*Z* = 2
Mo *K*α radiation
μ = 1.09 mm^−1^
*T* = 185 K
0.27 × 0.22 × 0.15 mm

#### Data collection

Bruker SMART 1000 CCD area-detector diffractometer
Absorption correction: multi-scan (*SADABS*; Bruker, 2001 ▶) *T*_min_ = 0.757, *T*_max_ = 0.853
13829 measured reflections
9496 independent reflections
8034 reflections with *I* > 2σ(*I*)
*R*_int_ = 0.016

#### Refinement

*R*[*F* ^2^ > 2σ(*F* ^2^)] = 0.037
*wR*(*F* ^2^) = 0.100
*S* = 1.04
9496 reflections
714 parameters
H-atom parameters constrained
Δρ_max_ = 0.39 e Å^−3^
Δρ_min_ = −0.66 e Å^−3^

### 

Data collection: *SMART* (Bruker, 2007 ▶); cell refinement: *SAINT* (Bruker, 2007 ▶); data reduction: *SAINT*; program(s) used to solve structure: *SHELXTL* (Sheldrick, 2008 ▶); program(s) used to refine structure: *SHELXTL*; molecular graphics: *SHELXTL*; software used to prepare material for publication: *SHELXTL*.

## Supplementary Material

Crystal structure: contains datablock(s) global, I. DOI: 10.1107/S1600536812013396/xu5481sup1.cif

Structure factors: contains datablock(s) I. DOI: 10.1107/S1600536812013396/xu5481Isup2.hkl

Additional supplementary materials:  crystallographic information; 3D view; checkCIF report

## Figures and Tables

**Table 1:** Hydrogen-bond geometry (Å, °)

*D*—H⋯*A*	*D*—H	H⋯*A*	*D*⋯*A*	*D*—H⋯*A*
O8—H8*A*⋯O7^i^	0.85	1.97	2.821 (5)	179
O8—H8*B*⋯O6^ii^	0.85	2.10	2.952 (4)	179
C2—H2*A*⋯O5	0.99	2.46	3.356 (4)	151
C3—H3*B*⋯O5	0.99	2.55	3.425 (3)	147
C24—H24*A*⋯N7	0.99	2.52	3.382 (4)	145
C31—H31*B*⋯O2	0.99	2.30	3.259 (4)	163

Symmetry codes: (i) 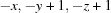; (ii) 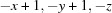.

**Fig. 1.**
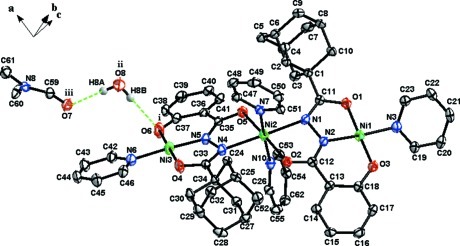
Molecular structure of the title compound. H atoms not involved in hydrogen bonds have been omitted for clarity. Symmetry codes: (i) -1+x, y, z; (ii) x, 1-y, -z; (iii) x, y, -1+z.

## supplementary crystallographic information

### Comment

Metal complexes of hydrazide and its ramification have been paid much attention to in recent years because of their prodigious applications in magnetism material, optical material, automatic recognition and assembly of molecules, catalysis and biochemistry, and so on (Alexiou *et al.*, 2003; Li *et al.*, 1996; Zeng *et al.*, 2007; Zhou *et al.*, 2010). *N*-acylsalicylhydrazide ligands, one of this type of ligand, which contain N, O coordination atoms, the great conjugated system and rich hydrogen-bonded donors and accepters, have been widely used to construct polynuclear complexes and coordination polymers with interesting structural motifs, such as the one-dimensional, the two-dimensional, the three-dimensional and the metallacrown(Liu *et al.*, 2008; Moon *et al.*, 2006; Qin *et al.*, 2011; Wang *et al.*, 2005). For nickel(II) complexes with *N*-acylsalicylhydrazide ligands, we can see that trinuclear complexes is more common from the former reports (Lin *et al.*, 2007); Meng *et al.*, 2007); Xiao & Jin, 2008); Yang & Lin, 2005) and most of them are unstable in air. We report here a new trinuclear nickel(II) complex, [Ni_3_(C_18_H_19_N_2_O_3_)_2_(py)_4_].DMF.H_2_O, which is stable at room temperature. Its molecular configuration was illustrated in Fig. 1.

In the molecular structure, the arrangement of three Ni^2+^ ions and the ligands which were coordinated to the central Ni^2+^ ion in axial positions are different from the reported trinuclear nickel(II) complexes containing *N*-acylsalicylhydrazide ligands (Yang & Lin, 2005; Xiao & Jin, 2008). In this complex, three Ni^2+^ ions are arranged in an arcuate shape. The central Ni2 atom adopts a distorted octahedral geometry and is coordinated by two hydrazide nitrogen atoms (N1, N4) and two salicyl carbonyl oxygen atoms (O2, O5) from two bridge deprotonated *N*-adamantanecarbonylsalicylichydrazide ligands (abbreviated as (ashz)^3-^) in the equatorial plane and by two nitrogen atoms (N7, N10) from twopyridine molecules in the axial positions. There is a reverse torsion to be occurred between the two planes of the bridge (ashz)^3-^ ligands because of the steric hindrance effect caused by adamantly. This torsion led the bond angles of O5—Ni2—N10 and O2—Ni2—N10 to be pressed to 85.958 (3)° and 84.284 (3)° respectively from the ideal 90°. Two other Ni^2+^ ions on two side adopt square-planar coordination environments and are coordinated respectively by a phenolic oxygen atom, a adamantanecarbonyl oxygen atom, a hydrazide nitrogen atom and a pyridine nitrogen atom. By O(8)—H(8 A)···O(7) and O(8)—H(8B)···O(6) hydrogen bonds, two hydrogen atoms of water molecule are respectively connected to the phenolic oxygen atom(O6) and the oxygen atom(O7) of DMF to forming dimethylformamide solvate monohydrate.

### Experimental

*Synthesis of ligand H_3_ashz*

Adamantanecarbonyl chloride (6.0 g, 0.03 mol) which was dissolved in tetrahydrofuran(30.0 ml)was dropped slowly into a solution of salicylhydrazide (5.5 g, 0.036 mol) and triethylamine(2.0 ml) dissolved in 60.0 ml of tetrahydrofuran at 0°C. After dropped off, the mixture was slowly warmed up to the room temperature, stirred continually for 24 h and then filtered. The filtrate was recrystallizated by distilled water, and the yellow product was obtained(yield 8.31 g, 87.5%). IR(*PE Spectrum One FT—IR Spectrometer*, KBr tablet, cm^-1^): 3352(*m*), 3243(*s*), 2915(*s*), 2851(*m*), 1665(*s*), 1637(*s*), 1608(*m*), 1531(*m*), 1515(*m*), 1495(*m*), 1456(*m*). 1278(*m*), 1236(*s*), 749(*m*), 709(*m*). ESI-Ms: M—H¯ peak at m/*z* 313.01.

*Synthesis of the complex*

[Ni_3_(C_18_H_19_N_2_O_3_)_2_(py)_4_].DMF.H_2_O Ni(OAc)_2_.4H_2_O(0.15 mmol), H_3_ashz (0.10 mmol) and five drops of pyridine were mixed into the solution of DMF(2 ml) and acetonitrile (10 ml). Stirring for 10 min, the resulting solution was filtered and left to stand at room temperature. Red rhomboid crystals suitable for X-ray analysis were obtained (yield 52.1%) by slowly volatilizing the solvent over a period of two weeks. Carbon, hydrogen and nitrogen were determined by a Perkin-Elmer 2400II CHN element analysis instrument. Analysis, calculated for [Ni_3_(C_18_H_19_N_2_O_3_)_2_(py)_4_].DMF.H_2_O: C, 58.74%: H, 5.60%; N, 10.44%. found: C, 58.87%; H, 5.60%; N, 10.53%. IR(KBr tablet, cm^-1^): 3468(w), 3071(w), 2903(*s*), 2840(*m*), 1670(*m*), 1597(*s*), 1569(*s*), 1503(*s*), 1481(*s*), 1443(*s*), 1403(*s*), 1334(*m*), 1305(*m*), 1264(*m*), 753(*s*), 693(*s*).

### Refinement

H atoms were placed in geometrically calculated positions and refined as riding atoms, with O—H = 0.85 and C—H = 0.95–1.00 Å and Uiso(H) = 1.5U_eq_(C) for methyl H atoms and 1.2U_eq_(C,O) for the others.

### Crystal data

**Table:**

[Ni_3_(C_18_H_19_N_2_O_3_)_2_(C_5_H_5_N)_4_]·C_3_H_7_NO·H_2_O	*Z* = 2
*M**_r_* = 1206.35	*F*(000) = 1264
Triclinic, *P*1	*D*_x_ = 1.471 Mg m^−^^3^
Hall symbol: -P 1	Mo *K*α radiation, λ = 0.71073 Å
*a* = 14.3496 (8) Å	Cell parameters from 6088 reflections
*b* = 14.8499 (9) Å	θ = 2.2–26.0°
*c* = 15.2256 (9) Å	µ = 1.09 mm^−^^1^
α = 62.061 (1)°	*T* = 185 K
β = 72.261 (1)°	Block, red
γ = 85.202 (1)°	0.27 × 0.22 × 0.15 mm
*V* = 2723.3 (3) Å^3^	

### Data collection

**Table:**

Bruker SMART 1000 CCD area-detector diffractometer	9496 independent reflections
Radiation source: fine-focus sealed tube	8034 reflections with *I* > 2σ(*I*)
Graphite monochromator	*R*_int_ = 0.016
φ and ω scans	θ_max_ = 25.1°, θ_min_ = 1.6°
Absorption correction: multi-scan (*SADABS*; Bruker, 2001)	*h* = −16→17
*T*_min_ = 0.757, *T*_max_ = 0.853	*k* = −17→17
13829 measured reflections	*l* = −15→18

### Refinement

**Table:**

Refinement on *F*^2^	Primary atom site location: structure-invariant direct methods
Least-squares matrix: full	Secondary atom site location: difference Fourier map
*R*[*F*^2^ > 2σ(*F*^2^)] = 0.037	Hydrogen site location: inferred from neighbouring sites
*wR*(*F*^2^) = 0.100	H-atom parameters constrained
*S* = 1.04	*w* = 1/[σ^2^(*F*_o_^2^) + (0.0519*P*)^2^ + 1.7341*P*] where *P* = (*F*_o_^2^ + 2*F*_c_^2^)/3
9496 reflections	(Δ/σ)_max_ < 0.001
714 parameters	Δρ_max_ = 0.39 e Å^−^^3^
0 restraints	Δρ_min_ = −0.66 e Å^−^^3^

### Special details

**Table:**

Geometry. All e.s.d.'s (except the e.s.d. in the dihedral angle between two l.s. planes) are estimated using the full covariance matrix. The cell e.s.d.'s are taken into account individually in the estimation of e.s.d.'s in distances, angles and torsion angles; correlations between e.s.d.'s in cell parameters are only used when they are defined by crystal symmetry. An approximate (isotropic) treatment of cell e.s.d.'s is used for estimating e.s.d.'s involving l.s. planes.
Refinement. Refinement of *F*^2^ against ALL reflections. The weighted *R*-factor *wR* and goodness of fit *S* are based on *F*^2^, conventional *R*-factors *R* are based on *F*, with *F* set to zero for negative *F*^2^. The threshold expression of *F*^2^ > σ(*F*^2^) is used only for calculating *R*-factors(gt) *etc*. and is not relevant to the choice of reflections for refinement. *R*-factors based on *F*^2^ are statistically about twice as large as those based on *F*, and *R*- factors based on ALL data will be even larger.

### Fractional atomic coordinates and isotropic or equivalent isotropic displacement parameters (Å^2^)

**Table:**

	*x*	*y*	*z*	*U*_iso_*/*U*_eq_	
C1	0.29062 (18)	0.3762 (2)	0.3952 (2)	0.0228 (6)	
C2	0.35980 (19)	0.4437 (2)	0.2850 (2)	0.0258 (6)	
H2A	0.3704	0.4078	0.2424	0.031*	
H2B	0.3294	0.5082	0.2507	0.031*	
C3	0.3398 (2)	0.2765 (2)	0.4466 (2)	0.0299 (6)	
H3A	0.2965	0.2322	0.5176	0.036*	
H3B	0.3501	0.2393	0.4053	0.036*	
C4	0.4391 (2)	0.3006 (3)	0.4531 (2)	0.0360 (7)	
H4	0.4703	0.2354	0.4863	0.043*	
C5	0.5064 (2)	0.3679 (3)	0.3430 (2)	0.0364 (7)	
H5A	0.5175	0.3315	0.3008	0.044*	
H5B	0.5706	0.3831	0.3466	0.044*	
C6	0.4585 (2)	0.4677 (2)	0.2919 (2)	0.0320 (7)	
H6	0.5026	0.5116	0.2201	0.038*	
C7	0.4238 (2)	0.3567 (3)	0.5181 (3)	0.0439 (8)	
H7A	0.4877	0.3716	0.5227	0.053*	
H7B	0.3811	0.3132	0.5897	0.053*	
C8	0.3763 (2)	0.4563 (3)	0.4670 (3)	0.0404 (8)	
H8	0.3661	0.4930	0.5096	0.048*	
C9	0.4435 (2)	0.5240 (3)	0.3569 (3)	0.0403 (8)	
H9A	0.4135	0.5891	0.3240	0.048*	
H9B	0.5076	0.5398	0.3604	0.048*	
C10	0.2769 (2)	0.4329 (2)	0.4610 (2)	0.0313 (7)	
H10A	0.2457	0.4975	0.4291	0.038*	
H10B	0.2331	0.3901	0.5323	0.038*	
C11	0.19072 (18)	0.3516 (2)	0.39238 (19)	0.0216 (5)	
C12	0.04791 (18)	0.30740 (19)	0.27002 (19)	0.0213 (5)	
C13	−0.05672 (18)	0.28752 (19)	0.2881 (2)	0.0223 (5)	
C14	−0.0812 (2)	0.2582 (2)	0.2221 (2)	0.0279 (6)	
H14	−0.0301	0.2530	0.1681	0.033*	
C15	−0.1763 (2)	0.2367 (2)	0.2332 (2)	0.0327 (7)	
H15	−0.1907	0.2155	0.1885	0.039*	
C16	−0.2518 (2)	0.2463 (2)	0.3109 (2)	0.0345 (7)	
H16	−0.3181	0.2333	0.3182	0.041*	
C17	−0.23028 (19)	0.2746 (2)	0.3770 (2)	0.0307 (6)	
H17	−0.2825	0.2814	0.4292	0.037*	
C18	−0.13288 (19)	0.2938 (2)	0.3693 (2)	0.0243 (6)	
C19	−0.1626 (2)	0.2900 (2)	0.6377 (2)	0.0325 (7)	
H19	−0.1903	0.2518	0.6150	0.039*	
C20	−0.2156 (2)	0.2941 (2)	0.7278 (2)	0.0362 (7)	
H20	−0.2779	0.2578	0.7673	0.043*	
C21	−0.1772 (2)	0.3516 (2)	0.7602 (2)	0.0364 (7)	
H21	−0.2131	0.3560	0.8216	0.044*	
C22	−0.0864 (2)	0.4021 (2)	0.7018 (2)	0.0324 (7)	
H22	−0.0590	0.4433	0.7215	0.039*	
C23	−0.0353 (2)	0.3926 (2)	0.6140 (2)	0.0279 (6)	
H23	0.0287	0.4253	0.5757	0.033*	
C24	0.2374 (2)	0.4137 (2)	−0.0965 (2)	0.0319 (6)	
H24A	0.2207	0.4227	−0.0331	0.038*	
H24B	0.3019	0.4507	−0.1430	0.038*	
C25	0.1590 (2)	0.4585 (2)	−0.1525 (2)	0.0350 (7)	
H25	0.1561	0.5329	−0.1721	0.042*	
C26	0.0597 (2)	0.4028 (2)	−0.0788 (2)	0.0360 (7)	
H26A	0.0083	0.4319	−0.1135	0.043*	
H26B	0.0429	0.4117	−0.0154	0.043*	
C27	0.0641 (2)	0.2892 (2)	−0.0488 (2)	0.0336 (7)	
H27	−0.0012	0.2529	−0.0009	0.040*	
C28	0.0900 (2)	0.2746 (3)	−0.1456 (2)	0.0392 (7)	
H28A	0.0385	0.3009	−0.1807	0.047*	
H28B	0.0937	0.2010	−0.1257	0.047*	
C29	0.1886 (2)	0.3319 (3)	−0.2198 (2)	0.0392 (8)	
H29	0.2049	0.3230	−0.2839	0.047*	
C30	0.2681 (2)	0.2875 (3)	−0.1657 (2)	0.0345 (7)	
H30A	0.3325	0.3236	−0.2136	0.041*	
H30B	0.2722	0.2143	−0.1476	0.041*	
C31	0.1426 (2)	0.2434 (2)	0.0066 (2)	0.0289 (6)	
H31A	0.1450	0.1698	0.0260	0.035*	
H31B	0.1256	0.2502	0.0712	0.035*	
C32	0.1844 (2)	0.4455 (3)	−0.2508 (2)	0.0413 (8)	
H32A	0.1341	0.4748	−0.2869	0.050*	
H32B	0.2486	0.4820	−0.2989	0.050*	
C33	0.32242 (19)	0.2535 (2)	−0.0119 (2)	0.0247 (6)	
C34	0.24377 (19)	0.2993 (2)	−0.0659 (2)	0.0243 (6)	
C35	0.42577 (18)	0.1992 (2)	0.1817 (2)	0.0222 (5)	
C36	0.50165 (19)	0.1339 (2)	0.2230 (2)	0.0264 (6)	
C37	0.5680 (2)	0.0860 (2)	0.1709 (2)	0.0331 (7)	
C38	0.6336 (2)	0.0212 (3)	0.2204 (3)	0.0467 (9)	
H38	0.6772	−0.0131	0.1871	0.056*	
C39	0.6363 (2)	0.0061 (3)	0.3159 (3)	0.0469 (9)	
H39	0.6819	−0.0372	0.3472	0.056*	
C40	0.5723 (2)	0.0543 (3)	0.3662 (3)	0.0407 (8)	
H40	0.5738	0.0445	0.4320	0.049*	
C41	0.5066 (2)	0.1165 (2)	0.3199 (2)	0.0309 (6)	
H41	0.4628	0.1490	0.3551	0.037*	
C42	0.6283 (2)	0.0585 (2)	−0.0914 (3)	0.0362 (7)	
H42	0.6447	0.0297	−0.0281	0.043*	
C43	0.6803 (2)	0.0324 (3)	−0.1681 (3)	0.0433 (8)	
H43	0.7317	−0.0124	−0.1578	0.052*	
C44	0.6563 (2)	0.0725 (3)	−0.2593 (3)	0.0464 (9)	
H44	0.6907	0.0555	−0.3130	0.056*	
C45	0.5820 (2)	0.1375 (3)	−0.2717 (3)	0.0477 (9)	
H45	0.5640	0.1662	−0.3341	0.057*	
C46	0.5337 (2)	0.1605 (3)	−0.1918 (3)	0.0407 (8)	
H46	0.4822	0.2054	−0.2009	0.049*	
C47	0.37565 (19)	0.4790 (2)	0.0152 (2)	0.0283 (6)	
H47	0.4239	0.4310	0.0160	0.034*	
C48	0.3986 (2)	0.5787 (2)	−0.0627 (2)	0.0339 (7)	
H48	0.4616	0.5988	−0.1131	0.041*	
C49	0.3285 (2)	0.6488 (2)	−0.0661 (2)	0.0358 (7)	
H49	0.3420	0.7178	−0.1192	0.043*	
C50	0.2380 (2)	0.6164 (2)	0.0096 (2)	0.0330 (7)	
H50	0.1880	0.6626	0.0091	0.040*	
C51	0.2220 (2)	0.5157 (2)	0.0856 (2)	0.0269 (6)	
H51	0.1601	0.4942	0.1380	0.032*	
C52	0.1332 (2)	0.0861 (2)	0.2905 (2)	0.0290 (6)	
H52	0.1220	0.1183	0.2243	0.035*	
C53	0.2090 (2)	0.0869 (2)	0.4018 (2)	0.0327 (7)	
H53	0.2526	0.1194	0.4160	0.039*	
C54	0.1647 (2)	−0.0083 (2)	0.4786 (2)	0.0398 (8)	
H54	0.1775	−0.0398	0.5439	0.048*	
C55	0.0864 (2)	−0.0088 (2)	0.3630 (2)	0.0372 (7)	
H55	0.0441	−0.0408	0.3469	0.045*	
C59	−0.0459 (3)	0.0942 (4)	0.8286 (4)	0.0659 (11)	
H59	−0.0609	0.1617	0.7873	0.079*	
C60	0.0721 (4)	−0.0299 (4)	0.8530 (5)	0.115 (2)	
H60A	0.1205	−0.0217	0.8827	0.172*	
H60B	0.1021	−0.0607	0.8073	0.172*	
H60C	0.0158	−0.0744	0.9093	0.172*	
C61	0.1112 (3)	0.1364 (4)	0.6989 (3)	0.0745 (13)	
H61A	0.0816	0.2009	0.6660	0.112*	
H61B	0.1313	0.1063	0.6515	0.112*	
H61C	0.1686	0.1494	0.7144	0.112*	
N1	0.17739 (14)	0.33462 (16)	0.31992 (16)	0.0207 (5)	
N2	0.07407 (14)	0.31891 (17)	0.34132 (16)	0.0209 (5)	
N3	−0.07305 (16)	0.33827 (18)	0.58081 (17)	0.0264 (5)	
N4	0.32325 (15)	0.25005 (17)	0.07580 (17)	0.0224 (5)	
N5	0.40790 (15)	0.19873 (17)	0.10142 (17)	0.0238 (5)	
N6	0.55583 (16)	0.12256 (19)	−0.10201 (19)	0.0311 (5)	
N7	0.28944 (15)	0.44640 (17)	0.08939 (16)	0.0217 (5)	
N8	0.0404 (2)	0.0667 (2)	0.7946 (3)	0.0532 (8)	
C62	0.1021 (2)	−0.0562 (2)	0.4588 (2)	0.0390 (7)	
H62	0.0701	−0.1211	0.5105	0.047*	
N10	0.19352 (16)	0.13518 (17)	0.30863 (17)	0.0255 (5)	
Ni1	−0.00220 (2)	0.32886 (3)	0.45606 (3)	0.02189 (10)	
Ni2	0.24878 (2)	0.29225 (3)	0.19742 (2)	0.01995 (9)	
Ni3	0.48439 (2)	0.15812 (3)	0.00729 (3)	0.02747 (10)	
O1	0.11573 (13)	0.34834 (15)	0.46962 (14)	0.0266 (4)	
O2	0.11103 (12)	0.31102 (14)	0.18823 (13)	0.0220 (4)	
O3	−0.11911 (13)	0.31607 (15)	0.43894 (14)	0.0279 (4)	
O4	0.39292 (14)	0.21619 (16)	−0.06013 (15)	0.0320 (5)	
O5	0.37708 (12)	0.24968 (14)	0.22717 (14)	0.0252 (4)	
O6	0.57437 (14)	0.09919 (18)	0.07684 (17)	0.0400 (5)	
O7	−0.1092 (2)	0.0409 (3)	0.9089 (3)	0.0913 (11)	
O8	0.2154 (3)	0.8318 (3)	0.0105 (3)	0.1010 (12)	
H8B	0.2758	0.8522	−0.0146	0.121*	
H8A	0.1839	0.8705	0.0345	0.121*	

### Atomic displacement parameters (Å^2^)

**Table:**

	*U*^11^	*U*^22^	*U*^33^	*U*^12^	*U*^13^	*U*^23^
C1	0.0212 (13)	0.0271 (14)	0.0230 (14)	0.0013 (11)	−0.0071 (11)	−0.0137 (12)
C2	0.0239 (14)	0.0304 (15)	0.0240 (14)	0.0002 (12)	−0.0054 (11)	−0.0141 (12)
C3	0.0283 (15)	0.0338 (16)	0.0276 (15)	0.0035 (12)	−0.0102 (12)	−0.0134 (13)
C4	0.0278 (15)	0.0461 (19)	0.0400 (17)	0.0085 (14)	−0.0185 (14)	−0.0206 (15)
C5	0.0231 (14)	0.052 (2)	0.0422 (18)	0.0041 (14)	−0.0113 (13)	−0.0275 (16)
C6	0.0217 (14)	0.0419 (18)	0.0324 (16)	−0.0059 (13)	−0.0032 (12)	−0.0190 (14)
C7	0.0322 (17)	0.070 (2)	0.0373 (18)	−0.0011 (16)	−0.0166 (14)	−0.0273 (18)
C8	0.0330 (16)	0.063 (2)	0.0461 (19)	−0.0016 (15)	−0.0109 (14)	−0.0418 (18)
C9	0.0295 (16)	0.050 (2)	0.053 (2)	−0.0046 (14)	−0.0106 (15)	−0.0339 (17)
C10	0.0290 (15)	0.0447 (18)	0.0298 (15)	0.0033 (13)	−0.0089 (12)	−0.0251 (14)
C11	0.0216 (13)	0.0208 (13)	0.0209 (13)	0.0021 (11)	−0.0063 (11)	−0.0086 (11)
C12	0.0233 (13)	0.0176 (13)	0.0218 (13)	0.0041 (10)	−0.0086 (11)	−0.0076 (11)
C13	0.0197 (13)	0.0199 (13)	0.0242 (14)	0.0019 (10)	−0.0082 (11)	−0.0069 (11)
C14	0.0265 (14)	0.0333 (16)	0.0276 (15)	0.0056 (12)	−0.0108 (12)	−0.0162 (13)
C15	0.0301 (15)	0.0392 (17)	0.0367 (17)	0.0019 (13)	−0.0152 (13)	−0.0209 (14)
C16	0.0242 (15)	0.0390 (18)	0.0428 (18)	0.0020 (13)	−0.0132 (13)	−0.0192 (15)
C17	0.0203 (14)	0.0358 (17)	0.0334 (16)	0.0045 (12)	−0.0054 (12)	−0.0161 (13)
C18	0.0237 (13)	0.0225 (14)	0.0243 (14)	0.0039 (11)	−0.0074 (11)	−0.0092 (11)
C19	0.0285 (15)	0.0390 (17)	0.0290 (15)	−0.0002 (13)	−0.0028 (12)	−0.0182 (14)
C20	0.0287 (15)	0.0429 (18)	0.0281 (16)	0.0012 (14)	0.0008 (12)	−0.0151 (14)
C21	0.0401 (17)	0.0429 (18)	0.0271 (16)	0.0132 (15)	−0.0061 (13)	−0.0212 (14)
C22	0.0404 (17)	0.0335 (16)	0.0318 (16)	0.0125 (14)	−0.0155 (14)	−0.0209 (14)
C23	0.0280 (14)	0.0295 (15)	0.0271 (15)	0.0044 (12)	−0.0099 (12)	−0.0134 (13)
C24	0.0346 (16)	0.0306 (16)	0.0338 (16)	0.0027 (13)	−0.0130 (13)	−0.0161 (13)
C25	0.0460 (18)	0.0265 (16)	0.0359 (17)	0.0062 (14)	−0.0204 (14)	−0.0130 (13)
C26	0.0341 (16)	0.0458 (19)	0.0361 (17)	0.0154 (14)	−0.0194 (14)	−0.0220 (15)
C27	0.0298 (15)	0.0387 (17)	0.0314 (16)	−0.0002 (13)	−0.0119 (13)	−0.0135 (14)
C28	0.0503 (19)	0.0368 (18)	0.0417 (18)	0.0061 (15)	−0.0286 (16)	−0.0187 (15)
C29	0.0503 (19)	0.049 (2)	0.0259 (16)	0.0110 (16)	−0.0180 (14)	−0.0207 (15)
C30	0.0394 (17)	0.0446 (19)	0.0255 (15)	0.0098 (14)	−0.0113 (13)	−0.0212 (14)
C31	0.0304 (15)	0.0310 (16)	0.0247 (14)	0.0017 (12)	−0.0098 (12)	−0.0116 (12)
C32	0.0472 (19)	0.0436 (19)	0.0267 (16)	0.0047 (15)	−0.0159 (14)	−0.0088 (14)
C33	0.0221 (13)	0.0252 (14)	0.0263 (14)	0.0023 (11)	−0.0045 (11)	−0.0135 (12)
C34	0.0270 (14)	0.0288 (15)	0.0219 (14)	0.0051 (12)	−0.0085 (11)	−0.0153 (12)
C35	0.0189 (13)	0.0202 (13)	0.0252 (14)	0.0000 (10)	−0.0044 (11)	−0.0098 (11)
C36	0.0196 (13)	0.0234 (14)	0.0349 (16)	0.0023 (11)	−0.0085 (12)	−0.0126 (12)
C37	0.0264 (15)	0.0354 (17)	0.0438 (18)	0.0089 (13)	−0.0142 (13)	−0.0225 (14)
C38	0.0337 (17)	0.054 (2)	0.064 (2)	0.0227 (16)	−0.0216 (17)	−0.0357 (19)
C39	0.0396 (18)	0.046 (2)	0.060 (2)	0.0202 (16)	−0.0303 (17)	−0.0215 (18)
C40	0.0344 (17)	0.046 (2)	0.0403 (18)	0.0066 (15)	−0.0181 (14)	−0.0152 (15)
C41	0.0263 (14)	0.0329 (16)	0.0322 (16)	0.0049 (12)	−0.0104 (12)	−0.0136 (13)
C42	0.0305 (16)	0.0313 (17)	0.0423 (18)	0.0044 (13)	−0.0026 (13)	−0.0190 (14)
C43	0.0399 (18)	0.0385 (19)	0.051 (2)	0.0077 (15)	−0.0027 (16)	−0.0281 (17)
C44	0.0412 (19)	0.052 (2)	0.052 (2)	−0.0002 (16)	0.0056 (16)	−0.0393 (18)
C45	0.0409 (19)	0.065 (2)	0.045 (2)	0.0048 (17)	−0.0049 (16)	−0.0367 (19)
C46	0.0296 (16)	0.052 (2)	0.0433 (19)	0.0060 (15)	−0.0043 (14)	−0.0289 (17)
C47	0.0227 (14)	0.0369 (17)	0.0275 (15)	0.0014 (12)	−0.0081 (12)	−0.0163 (13)
C48	0.0263 (15)	0.0441 (18)	0.0266 (15)	−0.0067 (13)	−0.0061 (12)	−0.0125 (14)
C49	0.0414 (17)	0.0292 (16)	0.0310 (16)	−0.0070 (14)	−0.0132 (14)	−0.0068 (13)
C50	0.0370 (16)	0.0272 (16)	0.0342 (16)	0.0054 (13)	−0.0110 (13)	−0.0142 (13)
C51	0.0300 (15)	0.0270 (15)	0.0255 (14)	0.0031 (12)	−0.0059 (12)	−0.0156 (12)
C52	0.0335 (15)	0.0264 (15)	0.0276 (15)	0.0029 (12)	−0.0098 (12)	−0.0127 (12)
C53	0.0384 (16)	0.0301 (16)	0.0309 (16)	0.0036 (13)	−0.0137 (13)	−0.0135 (13)
C54	0.056 (2)	0.0329 (17)	0.0248 (16)	0.0079 (15)	−0.0145 (15)	−0.0082 (13)
C55	0.0380 (17)	0.0290 (16)	0.0439 (19)	−0.0007 (13)	−0.0125 (14)	−0.0158 (14)
C59	0.054 (2)	0.069 (3)	0.088 (3)	0.014 (2)	−0.023 (2)	−0.048 (3)
C60	0.072 (3)	0.061 (3)	0.136 (5)	0.023 (3)	−0.002 (3)	−0.006 (3)
C61	0.080 (3)	0.068 (3)	0.066 (3)	0.001 (2)	−0.013 (2)	−0.029 (2)
N1	0.0151 (10)	0.0241 (12)	0.0219 (11)	0.0009 (9)	−0.0050 (9)	−0.0102 (9)
N2	0.0158 (10)	0.0261 (12)	0.0202 (11)	0.0003 (9)	−0.0043 (9)	−0.0109 (9)
N3	0.0254 (12)	0.0298 (13)	0.0240 (12)	0.0032 (10)	−0.0053 (10)	−0.0141 (10)
N4	0.0203 (11)	0.0229 (12)	0.0253 (12)	0.0070 (9)	−0.0084 (9)	−0.0121 (10)
N5	0.0178 (11)	0.0253 (12)	0.0294 (12)	0.0069 (9)	−0.0073 (9)	−0.0144 (10)
N6	0.0250 (12)	0.0323 (14)	0.0361 (14)	0.0027 (10)	−0.0028 (10)	−0.0200 (11)
N7	0.0230 (11)	0.0241 (12)	0.0195 (11)	0.0016 (9)	−0.0064 (9)	−0.0111 (9)
N8	0.0508 (18)	0.0444 (18)	0.072 (2)	0.0105 (15)	−0.0221 (16)	−0.0325 (17)
C62	0.0445 (18)	0.0221 (15)	0.0374 (18)	0.0008 (13)	−0.0065 (14)	−0.0068 (13)
N10	0.0253 (12)	0.0252 (12)	0.0243 (12)	0.0058 (10)	−0.0068 (10)	−0.0114 (10)
Ni1	0.01830 (17)	0.0270 (2)	0.02113 (18)	0.00131 (14)	−0.00339 (14)	−0.01332 (15)
Ni2	0.01796 (17)	0.02193 (18)	0.02015 (18)	0.00337 (13)	−0.00538 (13)	−0.01051 (14)
Ni3	0.02173 (18)	0.0325 (2)	0.0326 (2)	0.00796 (15)	−0.00624 (15)	−0.02078 (17)
O1	0.0220 (9)	0.0373 (11)	0.0223 (10)	−0.0003 (8)	−0.0033 (8)	−0.0171 (9)
O2	0.0203 (9)	0.0279 (10)	0.0191 (9)	0.0030 (8)	−0.0062 (7)	−0.0122 (8)
O3	0.0195 (9)	0.0387 (12)	0.0287 (10)	0.0031 (8)	−0.0049 (8)	−0.0199 (9)
O4	0.0289 (10)	0.0426 (12)	0.0322 (11)	0.0137 (9)	−0.0097 (9)	−0.0252 (10)
O5	0.0221 (9)	0.0299 (10)	0.0289 (10)	0.0070 (8)	−0.0092 (8)	−0.0178 (9)
O6	0.0298 (11)	0.0536 (14)	0.0497 (14)	0.0211 (10)	−0.0163 (10)	−0.0348 (12)
O7	0.0562 (19)	0.088 (2)	0.124 (3)	0.0048 (18)	−0.004 (2)	−0.058 (2)
O8	0.092 (3)	0.090 (3)	0.104 (3)	0.002 (2)	−0.002 (2)	−0.048 (2)

### Geometric parameters (Å, º)

**Table:**

C1—C11	1.526 (3)	C32—H32B	0.9900
C1—C3	1.542 (4)	C33—O4	1.306 (3)
C1—C2	1.544 (4)	C33—N4	1.315 (3)
C1—C10	1.548 (4)	C33—C34	1.525 (4)
C2—C6	1.535 (4)	C35—O5	1.278 (3)
C2—H2A	0.9900	C35—N5	1.326 (3)
C2—H2B	0.9900	C35—C36	1.480 (3)
C3—C4	1.540 (4)	C36—C41	1.397 (4)
C3—H3A	0.9900	C36—C37	1.413 (4)
C3—H3B	0.9900	C37—O6	1.326 (4)
C4—C7	1.528 (4)	C37—C38	1.409 (4)
C4—C5	1.531 (4)	C38—C39	1.375 (5)
C4—H4	1.0000	C38—H38	0.9500
C5—C6	1.532 (4)	C39—C40	1.385 (5)
C5—H5A	0.9900	C39—H39	0.9500
C5—H5B	0.9900	C40—C41	1.374 (4)
C6—C9	1.529 (4)	C40—H40	0.9500
C6—H6	1.0000	C41—H41	0.9500
C7—C8	1.528 (5)	C42—N6	1.343 (4)
C7—H7A	0.9900	C42—C43	1.384 (4)
C7—H7B	0.9900	C42—H42	0.9500
C8—C9	1.533 (5)	C43—C44	1.373 (5)
C8—C10	1.535 (4)	C43—H43	0.9500
C8—H8	1.0000	C44—C45	1.374 (5)
C9—H9A	0.9900	C44—H44	0.9500
C9—H9B	0.9900	C45—C46	1.383 (4)
C10—H10A	0.9900	C45—H45	0.9500
C10—H10B	0.9900	C46—N6	1.342 (4)
C11—N1	1.310 (3)	C46—H46	0.9500
C11—O1	1.314 (3)	C47—N7	1.333 (3)
C12—O2	1.278 (3)	C47—C48	1.381 (4)
C12—N2	1.335 (3)	C47—H47	0.9500
C12—C13	1.471 (3)	C48—C49	1.380 (4)
C13—C14	1.404 (4)	C48—H48	0.9500
C13—C18	1.414 (4)	C49—C50	1.385 (4)
C14—C15	1.369 (4)	C49—H49	0.9500
C14—H14	0.9500	C50—C51	1.379 (4)
C15—C16	1.394 (4)	C50—H50	0.9500
C15—H15	0.9500	C51—N7	1.346 (3)
C16—C17	1.375 (4)	C51—H51	0.9500
C16—H16	0.9500	C52—N10	1.341 (4)
C17—C18	1.408 (4)	C52—C55	1.380 (4)
C17—H17	0.9500	C52—H52	0.9500
C18—O3	1.320 (3)	C53—N10	1.338 (4)
C19—N3	1.346 (4)	C53—C54	1.384 (4)
C19—C20	1.378 (4)	C53—H53	0.9500
C19—H19	0.9500	C54—C62	1.370 (5)
C20—C21	1.382 (4)	C54—H54	0.9500
C20—H20	0.9500	C55—C62	1.374 (4)
C21—C22	1.373 (4)	C55—H55	0.9500
C21—H21	0.9500	C59—O7	1.217 (5)
C22—C23	1.380 (4)	C59—N8	1.301 (5)
C22—H22	0.9500	C59—H59	0.9500
C23—N3	1.350 (3)	C60—N8	1.413 (5)
C23—H23	0.9500	C60—H60A	0.9800
C24—C25	1.537 (4)	C60—H60B	0.9800
C24—C34	1.540 (4)	C60—H60C	0.9800
C24—H24A	0.9900	C61—N8	1.445 (5)
C24—H24B	0.9900	C61—H61A	0.9800
C25—C26	1.526 (4)	C61—H61B	0.9800
C25—C32	1.529 (4)	C61—H61C	0.9800
C25—H25	1.0000	N1—N2	1.432 (3)
C26—C27	1.528 (4)	N1—Ni2	2.180 (2)
C26—H26A	0.9900	N2—Ni1	1.822 (2)
C26—H26B	0.9900	N3—Ni1	1.926 (2)
C27—C28	1.520 (4)	N4—N5	1.437 (3)
C27—C31	1.535 (4)	N4—Ni2	2.176 (2)
C27—H27	1.0000	N5—Ni3	1.834 (2)
C28—C29	1.526 (5)	N6—Ni3	1.947 (2)
C28—H28A	0.9900	N7—Ni2	2.097 (2)
C28—H28B	0.9900	C62—H62	0.9500
C29—C32	1.525 (5)	N10—Ni2	2.174 (2)
C29—C30	1.535 (4)	Ni1—O3	1.8124 (18)
C29—H29	1.0000	Ni1—O1	1.8277 (18)
C30—C34	1.542 (4)	Ni2—O5	2.0099 (17)
C30—H30A	0.9900	Ni2—O2	2.0109 (17)
C30—H30B	0.9900	Ni3—O4	1.8226 (19)
C31—C34	1.543 (4)	Ni3—O6	1.825 (2)
C31—H31A	0.9900	O8—H8B	0.8500
C31—H31B	0.9900	O8—H8A	0.8500
C32—H32A	0.9900		
			
C11—C1—C3	110.0 (2)	H32A—C32—H32B	108.3
C11—C1—C2	111.9 (2)	O4—C33—N4	120.3 (2)
C3—C1—C2	108.5 (2)	O4—C33—C34	114.5 (2)
C11—C1—C10	109.4 (2)	N4—C33—C34	125.3 (2)
C3—C1—C10	108.2 (2)	C33—C34—C24	111.4 (2)
C2—C1—C10	108.7 (2)	C33—C34—C30	109.7 (2)
C6—C2—C1	110.0 (2)	C24—C34—C30	108.4 (2)
C6—C2—H2A	109.7	C33—C34—C31	110.3 (2)
C1—C2—H2A	109.7	C24—C34—C31	108.6 (2)
C6—C2—H2B	109.7	C30—C34—C31	108.4 (2)
C1—C2—H2B	109.7	O5—C35—N5	122.6 (2)
H2A—C2—H2B	108.2	O5—C35—C36	119.3 (2)
C4—C3—C1	110.3 (2)	N5—C35—C36	118.0 (2)
C4—C3—H3A	109.6	C41—C36—C37	118.1 (2)
C1—C3—H3A	109.6	C41—C36—C35	118.4 (2)
C4—C3—H3B	109.6	C37—C36—C35	123.5 (2)
C1—C3—H3B	109.6	O6—C37—C38	117.0 (3)
H3A—C3—H3B	108.1	O6—C37—C36	124.5 (3)
C7—C4—C5	109.4 (3)	C38—C37—C36	118.5 (3)
C7—C4—C3	109.9 (2)	C39—C38—C37	121.7 (3)
C5—C4—C3	109.4 (2)	C39—C38—H38	119.2
C7—C4—H4	109.4	C37—C38—H38	119.2
C5—C4—H4	109.4	C38—C39—C40	119.8 (3)
C3—C4—H4	109.4	C38—C39—H39	120.1
C4—C5—C6	109.4 (2)	C40—C39—H39	120.1
C4—C5—H5A	109.8	C41—C40—C39	119.3 (3)
C6—C5—H5A	109.8	C41—C40—H40	120.3
C4—C5—H5B	109.8	C39—C40—H40	120.3
C6—C5—H5B	109.8	C40—C41—C36	122.6 (3)
H5A—C5—H5B	108.2	C40—C41—H41	118.7
C9—C6—C5	109.2 (2)	C36—C41—H41	118.7
C9—C6—C2	110.3 (2)	N6—C42—C43	123.3 (3)
C5—C6—C2	109.5 (2)	N6—C42—H42	118.3
C9—C6—H6	109.3	C43—C42—H42	118.3
C5—C6—H6	109.3	C44—C43—C42	118.8 (3)
C2—C6—H6	109.3	C44—C43—H43	120.6
C4—C7—C8	109.1 (2)	C42—C43—H43	120.6
C4—C7—H7A	109.9	C43—C44—C45	119.0 (3)
C8—C7—H7A	109.9	C43—C44—H44	120.5
C4—C7—H7B	109.9	C45—C44—H44	120.5
C8—C7—H7B	109.9	C44—C45—C46	118.9 (3)
H7A—C7—H7B	108.3	C44—C45—H45	120.6
C7—C8—C9	109.7 (3)	C46—C45—H45	120.6
C7—C8—C10	109.8 (3)	N6—C46—C45	123.3 (3)
C9—C8—C10	109.6 (2)	N6—C46—H46	118.3
C7—C8—H8	109.3	C45—C46—H46	118.3
C9—C8—H8	109.3	N7—C47—C48	123.6 (3)
C10—C8—H8	109.3	N7—C47—H47	118.2
C6—C9—C8	109.1 (3)	C48—C47—H47	118.2
C6—C9—H9A	109.9	C49—C48—C47	118.9 (3)
C8—C9—H9A	109.9	C49—C48—H48	120.6
C6—C9—H9B	109.9	C47—C48—H48	120.6
C8—C9—H9B	109.9	C48—C49—C50	118.6 (3)
H9A—C9—H9B	108.3	C48—C49—H49	120.7
C8—C10—C1	110.3 (2)	C50—C49—H49	120.7
C8—C10—H10A	109.6	C51—C50—C49	118.7 (3)
C1—C10—H10A	109.6	C51—C50—H50	120.7
C8—C10—H10B	109.6	C49—C50—H50	120.7
C1—C10—H10B	109.6	N7—C51—C50	123.3 (3)
H10A—C10—H10B	108.1	N7—C51—H51	118.3
N1—C11—O1	120.6 (2)	C50—C51—H51	118.3
N1—C11—C1	124.4 (2)	N10—C52—C55	123.3 (3)
O1—C11—C1	115.0 (2)	N10—C52—H52	118.3
O2—C12—N2	122.0 (2)	C55—C52—H52	118.3
O2—C12—C13	119.4 (2)	N10—C53—C54	123.2 (3)
N2—C12—C13	118.6 (2)	N10—C53—H53	118.4
C14—C13—C18	118.7 (2)	C54—C53—H53	118.4
C14—C13—C12	117.6 (2)	C62—C54—C53	118.9 (3)
C18—C13—C12	123.7 (2)	C62—C54—H54	120.5
C15—C14—C13	122.1 (3)	C53—C54—H54	120.5
C15—C14—H14	118.9	C62—C55—C52	118.9 (3)
C13—C14—H14	118.9	C62—C55—H55	120.6
C14—C15—C16	119.2 (3)	C52—C55—H55	120.6
C14—C15—H15	120.4	O7—C59—N8	125.6 (5)
C16—C15—H15	120.4	O7—C59—H59	117.2
C17—C16—C15	120.1 (3)	N8—C59—H59	117.2
C17—C16—H16	120.0	N8—C60—H60A	109.5
C15—C16—H16	120.0	N8—C60—H60B	109.5
C16—C17—C18	121.7 (3)	H60A—C60—H60B	109.5
C16—C17—H17	119.1	N8—C60—H60C	109.5
C18—C17—H17	119.1	H60A—C60—H60C	109.5
O3—C18—C17	117.5 (2)	H60B—C60—H60C	109.5
O3—C18—C13	124.5 (2)	N8—C61—H61A	109.5
C17—C18—C13	118.0 (2)	N8—C61—H61B	109.5
N3—C19—C20	122.1 (3)	H61A—C61—H61B	109.5
N3—C19—H19	118.9	N8—C61—H61C	109.5
C20—C19—H19	118.9	H61A—C61—H61C	109.5
C19—C20—C21	119.5 (3)	H61B—C61—H61C	109.5
C19—C20—H20	120.2	C11—N1—N2	108.00 (19)
C21—C20—H20	120.2	C11—N1—Ni2	144.34 (17)
C22—C21—C20	118.7 (3)	N2—N1—Ni2	106.17 (14)
C22—C21—H21	120.7	C12—N2—N1	115.3 (2)
C20—C21—H21	120.7	C12—N2—Ni1	129.67 (17)
C21—C22—C23	119.3 (3)	N1—N2—Ni1	114.78 (15)
C21—C22—H22	120.3	C19—N3—C23	118.0 (2)
C23—C22—H22	120.3	C19—N3—Ni1	120.22 (19)
N3—C23—C22	122.3 (3)	C23—N3—Ni1	121.75 (19)
N3—C23—H23	118.8	C33—N4—N5	107.8 (2)
C22—C23—H23	118.8	C33—N4—Ni2	145.27 (17)
C25—C24—C34	110.4 (2)	N5—N4—Ni2	106.93 (14)
C25—C24—H24A	109.6	C35—N5—N4	115.3 (2)
C34—C24—H24A	109.6	C35—N5—Ni3	129.29 (17)
C25—C24—H24B	109.6	N4—N5—Ni3	114.77 (15)
C34—C24—H24B	109.6	C46—N6—C42	116.7 (3)
H24A—C24—H24B	108.1	C46—N6—Ni3	121.03 (19)
C26—C25—C32	109.7 (3)	C42—N6—Ni3	122.3 (2)
C26—C25—C24	108.9 (2)	C47—N7—C51	117.0 (2)
C32—C25—C24	110.0 (2)	C47—N7—Ni2	123.50 (18)
C26—C25—H25	109.4	C51—N7—Ni2	119.06 (18)
C32—C25—H25	109.4	C59—N8—C60	121.8 (4)
C24—C25—H25	109.4	C59—N8—C61	121.4 (4)
C25—C26—C27	109.4 (2)	C60—N8—C61	116.7 (3)
C25—C26—H26A	109.8	C54—C62—C55	118.9 (3)
C27—C26—H26A	109.8	C54—C62—H62	120.6
C25—C26—H26B	109.8	C55—C62—H62	120.6
C27—C26—H26B	109.8	C53—N10—C52	116.8 (2)
H26A—C26—H26B	108.2	C53—N10—Ni2	122.35 (19)
C28—C27—C26	110.2 (2)	C52—N10—Ni2	120.49 (18)
C28—C27—C31	108.6 (2)	O3—Ni1—N2	96.43 (8)
C26—C27—C31	110.0 (2)	O3—Ni1—O1	177.27 (9)
C28—C27—H27	109.3	N2—Ni1—O1	83.60 (8)
C26—C27—H27	109.3	O3—Ni1—N3	88.29 (9)
C31—C27—H27	109.3	N2—Ni1—N3	175.20 (9)
C27—C28—C29	109.4 (2)	O1—Ni1—N3	91.73 (9)
C27—C28—H28A	109.8	O5—Ni2—O2	170.13 (8)
C29—C28—H28A	109.8	O5—Ni2—N7	99.08 (8)
C27—C28—H28B	109.8	O2—Ni2—N7	90.67 (8)
C29—C28—H28B	109.8	O5—Ni2—N10	85.96 (8)
H28A—C28—H28B	108.2	O2—Ni2—N10	84.28 (8)
C28—C29—C32	110.5 (3)	N7—Ni2—N10	174.94 (8)
C28—C29—C30	109.0 (3)	O5—Ni2—N4	78.18 (7)
C32—C29—C30	109.6 (3)	O2—Ni2—N4	103.63 (7)
C28—C29—H29	109.2	N7—Ni2—N4	89.49 (8)
C32—C29—H29	109.2	N10—Ni2—N4	91.97 (8)
C30—C29—H29	109.2	O5—Ni2—N1	100.54 (7)
C29—C30—C34	110.1 (2)	O2—Ni2—N1	77.70 (7)
C29—C30—H30A	109.6	N7—Ni2—N1	90.38 (8)
C34—C30—H30A	109.6	N10—Ni2—N1	88.28 (8)
C29—C30—H30B	109.6	N4—Ni2—N1	178.67 (8)
C34—C30—H30B	109.6	O4—Ni3—O6	179.06 (9)
H30A—C30—H30B	108.2	O4—Ni3—N5	83.07 (9)
C27—C31—C34	110.0 (2)	O6—Ni3—N5	96.18 (9)
C27—C31—H31A	109.7	O4—Ni3—N6	90.55 (9)
C34—C31—H31A	109.7	O6—Ni3—N6	90.20 (9)
C27—C31—H31B	109.7	N5—Ni3—N6	173.61 (10)
C34—C31—H31B	109.7	C11—O1—Ni1	112.84 (16)
H31A—C31—H31B	108.2	C12—O2—Ni2	113.97 (15)
C29—C32—C25	108.9 (2)	C18—O3—Ni1	126.21 (16)
C29—C32—H32A	109.9	C33—O4—Ni3	114.06 (17)
C25—C32—H32A	109.9	C35—O5—Ni2	114.09 (15)
C29—C32—H32B	109.9	C37—O6—Ni3	125.48 (17)
C25—C32—H32B	109.9	H8B—O8—H8A	108.4
			
C11—C1—C2—C6	179.1 (2)	O4—C33—N4—N5	−1.7 (3)
C3—C1—C2—C6	−59.3 (3)	C34—C33—N4—N5	178.5 (2)
C10—C1—C2—C6	58.1 (3)	O4—C33—N4—Ni2	177.3 (2)
C11—C1—C3—C4	−178.1 (2)	C34—C33—N4—Ni2	−2.5 (5)
C2—C1—C3—C4	59.1 (3)	O5—C35—N5—N4	−6.4 (4)
C10—C1—C3—C4	−58.6 (3)	C36—C35—N5—N4	170.3 (2)
C1—C3—C4—C7	60.1 (3)	O5—C35—N5—Ni3	164.11 (19)
C1—C3—C4—C5	−60.0 (3)	C36—C35—N5—Ni3	−19.2 (4)
C7—C4—C5—C6	−60.5 (3)	C33—N4—N5—C35	172.6 (2)
C3—C4—C5—C6	60.0 (3)	Ni2—N4—N5—C35	−6.8 (3)
C4—C5—C6—C9	60.5 (3)	C33—N4—N5—Ni3	0.7 (3)
C4—C5—C6—C2	−60.4 (3)	Ni2—N4—N5—Ni3	−178.72 (10)
C1—C2—C6—C9	−59.7 (3)	C45—C46—N6—C42	−0.6 (5)
C1—C2—C6—C5	60.5 (3)	C45—C46—N6—Ni3	−179.9 (3)
C5—C4—C7—C8	60.2 (3)	C43—C42—N6—C46	1.0 (4)
C3—C4—C7—C8	−59.9 (3)	C43—C42—N6—Ni3	−179.8 (2)
C4—C7—C8—C9	−60.4 (3)	C48—C47—N7—C51	0.9 (4)
C4—C7—C8—C10	60.1 (3)	C48—C47—N7—Ni2	172.9 (2)
C5—C6—C9—C8	−60.3 (3)	C50—C51—N7—C47	0.3 (4)
C2—C6—C9—C8	60.1 (3)	C50—C51—N7—Ni2	−172.0 (2)
C7—C8—C9—C6	60.5 (3)	O7—C59—N8—C60	−3.6 (7)
C10—C8—C9—C6	−60.1 (3)	O7—C59—N8—C61	−178.9 (4)
C7—C8—C10—C1	−60.3 (3)	C53—C54—C62—C55	0.8 (5)
C9—C8—C10—C1	60.2 (3)	C52—C55—C62—C54	−0.9 (4)
C11—C1—C10—C8	178.7 (2)	C54—C53—N10—C52	−1.3 (4)
C3—C1—C10—C8	58.9 (3)	C54—C53—N10—Ni2	171.8 (2)
C2—C1—C10—C8	−58.7 (3)	C55—C52—N10—C53	1.2 (4)
C3—C1—C11—N1	−84.0 (3)	C55—C52—N10—Ni2	−172.0 (2)
C2—C1—C11—N1	36.7 (3)	C12—N2—Ni1—O3	0.8 (2)
C10—C1—C11—N1	157.3 (2)	N1—N2—Ni1—O3	174.76 (17)
C3—C1—C11—O1	96.0 (3)	C12—N2—Ni1—O1	−176.5 (2)
C2—C1—C11—O1	−143.3 (2)	N1—N2—Ni1—O1	−2.50 (17)
C10—C1—C11—O1	−22.7 (3)	C12—N2—Ni1—N3	170.0 (11)
O2—C12—C13—C14	9.4 (4)	N1—N2—Ni1—N3	−16.0 (13)
N2—C12—C13—C14	−169.2 (2)	C19—N3—Ni1—O3	30.2 (2)
O2—C12—C13—C18	−172.4 (2)	C23—N3—Ni1—O3	−149.9 (2)
N2—C12—C13—C18	9.1 (4)	C19—N3—Ni1—N2	−139.1 (11)
C18—C13—C14—C15	0.8 (4)	C23—N3—Ni1—N2	40.8 (13)
C12—C13—C14—C15	179.2 (3)	C19—N3—Ni1—O1	−152.6 (2)
C13—C14—C15—C16	1.4 (5)	C23—N3—Ni1—O1	27.3 (2)
C14—C15—C16—C17	−1.6 (5)	C47—N7—Ni2—O5	40.5 (2)
C15—C16—C17—C18	−0.5 (5)	C51—N7—Ni2—O5	−147.63 (19)
C16—C17—C18—O3	−177.1 (3)	C47—N7—Ni2—O2	−141.0 (2)
C16—C17—C18—C13	2.7 (4)	C51—N7—Ni2—O2	30.81 (19)
C14—C13—C18—O3	176.9 (2)	C47—N7—Ni2—N10	−144.3 (9)
C12—C13—C18—O3	−1.3 (4)	C51—N7—Ni2—N10	27.6 (10)
C14—C13—C18—C17	−2.8 (4)	C47—N7—Ni2—N4	−37.4 (2)
C12—C13—C18—C17	178.9 (2)	C51—N7—Ni2—N4	134.43 (19)
N3—C19—C20—C21	1.6 (5)	C47—N7—Ni2—N1	141.3 (2)
C19—C20—C21—C22	−0.8 (4)	C51—N7—Ni2—N1	−46.89 (19)
C20—C21—C22—C23	−1.3 (4)	C53—N10—Ni2—O5	47.3 (2)
C21—C22—C23—N3	2.7 (4)	C52—N10—Ni2—O5	−139.8 (2)
C34—C24—C25—C26	60.8 (3)	C53—N10—Ni2—O2	−131.2 (2)
C34—C24—C25—C32	−59.5 (3)	C52—N10—Ni2—O2	41.68 (19)
C32—C25—C26—C27	60.0 (3)	C53—N10—Ni2—N7	−128.0 (9)
C24—C25—C26—C27	−60.5 (3)	C52—N10—Ni2—N7	44.9 (10)
C25—C26—C27—C28	−59.4 (3)	C53—N10—Ni2—N4	125.3 (2)
C25—C26—C27—C31	60.4 (3)	C52—N10—Ni2—N4	−61.8 (2)
C26—C27—C28—C29	58.6 (3)	C53—N10—Ni2—N1	−53.4 (2)
C31—C27—C28—C29	−62.0 (3)	C52—N10—Ni2—N1	119.5 (2)
C27—C28—C29—C32	−59.0 (3)	C33—N4—Ni2—O5	−167.6 (3)
C27—C28—C29—C30	61.6 (3)	N5—N4—Ni2—O5	11.41 (15)
C28—C29—C30—C34	−60.1 (3)	C33—N4—Ni2—O2	22.4 (3)
C32—C29—C30—C34	61.0 (3)	N5—N4—Ni2—O2	−158.64 (15)
C28—C27—C31—C34	61.3 (3)	C33—N4—Ni2—N7	−68.2 (3)
C26—C27—C31—C34	−59.5 (3)	N5—N4—Ni2—N7	110.80 (16)
C28—C29—C32—C25	59.6 (3)	C33—N4—Ni2—N10	107.0 (3)
C30—C29—C32—C25	−60.6 (3)	N5—N4—Ni2—N10	−74.04 (16)
C26—C25—C32—C29	−59.9 (3)	C33—N4—Ni2—N1	−152 (3)
C24—C25—C32—C29	59.9 (3)	N5—N4—Ni2—N1	27 (4)
O4—C33—C34—C24	−119.0 (3)	C11—N1—Ni2—O5	10.3 (3)
N4—C33—C34—C24	60.7 (3)	N2—N1—Ni2—O5	−152.61 (14)
O4—C33—C34—C30	1.0 (3)	C11—N1—Ni2—O2	−179.6 (3)
N4—C33—C34—C30	−179.3 (3)	N2—N1—Ni2—O2	17.47 (14)
O4—C33—C34—C31	120.3 (3)	C11—N1—Ni2—N7	−89.0 (3)
N4—C33—C34—C31	−59.9 (3)	N2—N1—Ni2—N7	108.08 (15)
C25—C24—C34—C33	179.0 (2)	C11—N1—Ni2—N10	95.9 (3)
C25—C24—C34—C30	58.3 (3)	N2—N1—Ni2—N10	−67.05 (15)
C25—C24—C34—C31	−59.4 (3)	C11—N1—Ni2—N4	−5 (4)
C29—C30—C34—C33	179.2 (2)	N2—N1—Ni2—N4	−168 (4)
C29—C30—C34—C24	−59.0 (3)	C35—N5—Ni3—O4	−170.3 (2)
C29—C30—C34—C31	58.7 (3)	N4—N5—Ni3—O4	0.24 (17)
C27—C31—C34—C33	−179.4 (2)	C35—N5—Ni3—O6	10.3 (3)
C27—C31—C34—C24	58.3 (3)	N4—N5—Ni3—O6	−179.17 (18)
C27—C31—C34—C30	−59.3 (3)	C35—N5—Ni3—N6	−166.5 (8)
O5—C35—C36—C41	10.3 (4)	N4—N5—Ni3—N6	4.0 (10)
N5—C35—C36—C41	−166.4 (3)	C46—N6—Ni3—O4	11.1 (2)
O5—C35—C36—C37	−171.3 (3)	C42—N6—Ni3—O4	−168.1 (2)
N5—C35—C36—C37	11.9 (4)	C46—N6—Ni3—O6	−169.5 (2)
C41—C36—C37—O6	−177.0 (3)	C42—N6—Ni3—O6	11.3 (2)
C35—C36—C37—O6	4.7 (5)	C46—N6—Ni3—N5	7.4 (10)
C41—C36—C37—C38	1.8 (4)	C42—N6—Ni3—N5	−171.8 (8)
C35—C36—C37—C38	−176.6 (3)	N1—C11—O1—Ni1	−4.6 (3)
O6—C37—C38—C39	176.9 (3)	C1—C11—O1—Ni1	175.37 (17)
C36—C37—C38—C39	−1.9 (5)	O3—Ni1—O1—C11	−87.1 (16)
C37—C38—C39—C40	1.0 (6)	N2—Ni1—O1—C11	3.74 (18)
C38—C39—C40—C41	0.2 (5)	N3—Ni1—O1—C11	−177.38 (18)
C39—C40—C41—C36	−0.3 (5)	N2—C12—O2—Ni2	17.6 (3)
C37—C36—C41—C40	−0.7 (4)	C13—C12—O2—Ni2	−160.86 (18)
C35—C36—C41—C40	177.8 (3)	O5—Ni2—O2—C12	61.6 (5)
N6—C42—C43—C44	−0.9 (5)	N7—Ni2—O2—C12	−109.35 (18)
C42—C43—C44—C45	0.3 (5)	N10—Ni2—O2—C12	70.37 (18)
C43—C44—C45—C46	0.0 (5)	N4—Ni2—O2—C12	161.02 (17)
C44—C45—C46—N6	0.1 (5)	N1—Ni2—O2—C12	−19.11 (17)
N7—C47—C48—C49	−1.4 (4)	C17—C18—O3—Ni1	172.16 (19)
C47—C48—C49—C50	0.6 (4)	C13—C18—O3—Ni1	−7.6 (4)
C48—C49—C50—C51	0.5 (4)	N2—Ni1—O3—C18	7.2 (2)
C49—C50—C51—N7	−1.0 (4)	O1—Ni1—O3—C18	97.7 (16)
N10—C53—C54—C62	0.3 (5)	N3—Ni1—O3—C18	−171.9 (2)
N10—C52—C55—C62	−0.1 (4)	N4—C33—O4—Ni3	2.1 (3)
O1—C11—N1—N2	2.5 (3)	C34—C33—O4—Ni3	−178.19 (18)
C1—C11—N1—N2	−177.5 (2)	O6—Ni3—O4—C33	37 (6)
O1—C11—N1—Ni2	−160.2 (2)	N5—Ni3—O4—C33	−1.17 (19)
C1—C11—N1—Ni2	19.8 (5)	N6—Ni3—O4—C33	179.2 (2)
O2—C12—N2—N1	−0.5 (3)	N5—C35—O5—Ni2	17.5 (3)
C13—C12—N2—N1	178.0 (2)	C36—C35—O5—Ni2	−159.12 (19)
O2—C12—N2—Ni1	173.44 (18)	O2—Ni2—O5—C35	86.3 (4)
C13—C12—N2—Ni1	−8.0 (4)	N7—Ni2—O5—C35	−102.88 (18)
C11—N1—N2—C12	175.6 (2)	N10—Ni2—O5—C35	77.54 (18)
Ni2—N1—N2—C12	−14.7 (2)	N4—Ni2—O5—C35	−15.33 (18)
C11—N1—N2—Ni1	0.8 (2)	N1—Ni2—O5—C35	165.03 (18)
Ni2—N1—N2—Ni1	170.38 (10)	C38—C37—O6—Ni3	167.5 (2)
C20—C19—N3—C23	−0.3 (4)	C36—C37—O6—Ni3	−13.8 (4)
C20—C19—N3—Ni1	179.6 (2)	O4—Ni3—O6—C37	−31 (6)
C22—C23—N3—C19	−1.8 (4)	N5—Ni3—O6—C37	6.7 (3)
C22—C23—N3—Ni1	178.2 (2)	N6—Ni3—O6—C37	−173.6 (3)

### Hydrogen-bond geometry (Å, º)

**Table:**

*D*—H···*A*	*D*—H	H···*A*	*D*···*A*	*D*—H···*A*
O8—H8*A*···O7^i^	0.85	1.97	2.821 (5)	179
O8—H8*B*···O6^ii^	0.85	2.10	2.952 (4)	179
C2—H2*A*···O5	0.99	2.46	3.356 (4)	151
C3—H3*B*···O5	0.99	2.55	3.425 (3)	147
C24—H24*A*···N7	0.99	2.52	3.382 (4)	145
C31—H31*B*···O2	0.99	2.30	3.259 (4)	163

Symmetry codes: (i) −*x*, −*y*+1, −*z*+1; (ii) −*x*+1, −*y*+1, −*z*.
